# Predictors of attrition from a weight loss program. A study of adult patients with obesity in a community setting

**DOI:** 10.1007/s40519-020-00990-9

**Published:** 2020-08-20

**Authors:** Valentina Ponzo, Elena Scumaci, Ilaria Goitre, Guglielmo Beccuti, Andrea Benso, Sara Belcastro, Chiara Crespi, Franco De Michieli, Marianna Pellegrini, Paola Scuntero, Enrica Marzola, Giovanni Abbate-Daga, Ezio Ghigo, Fabio Broglio, Simona Bo

**Affiliations:** 1 Department of Medical Sciences, University of Torino, c.so AM Dogliotti 14, 10126 Turin, Italy; 2 Center for the Treatment of Diabetes and Metabolic Diseases, “Città Della Salute E Della Scienza” Hospital of Torino, Turin, Italy; 3 Department of Neuroscience, University of Torino, Turin, Italy

**Keywords:** Obesity, Dropouts, Treatment failure, Weight management

## Abstract

**Purpose:**

Obesity unit attrition is frequent and contributes to treatment failure. Many studies evaluating attrition predictors were part of randomized trials, and different terminology and criteria were used in the engagement field. We aimed to investigate the factors potentially implicated in early (< 12 weeks) and late (> 12 weeks) attrition from an obesity unit in a community setting

**Methods:**

This was a retrospective cohort study of 250 patients with obesity who were followed-up at our obesity unit. Our program included at least 6 meetings in 12 months. Sociodemographic and anthropometric data, and psychometric questionnaires were collected from all participants.

**Results:**

One-hundred thirty-four (53.6%) participants dropped out. Those individuals showed lower BMI, lower overall health status, and increased depression scores. In a multiple regression model, BMI (inversely; OR = 0.90; 95%CI 0.84–0.96) and depression score (directly, OR = 1.05; 1.00–1.10) were associated with attrition risk. Early dropouts (*n *= 47) had lower weights, smaller waist circumferences and worse mental health scores than late dropouts (*n* = 87) and more frequently lived alone. When compared to completers, early dropouts had lower weights, BMIs, waist circumferences, overall health and mental status scores, increased depression scores and percentage of individuals living alone. In a multiple regression, lower BMI (OR = 0.83; 0.75–0.92), lower mental status score (OR = 3.17; 1.17–8.59) and living alone (OR = 2.25; 1.02–4.97) were associated with early attrition risk.

**Conclusion:**

Lower BMI and increased depression score were associated with attrition. Early attrition was associated with lower weight, decreased mental well-being, and living alone. Individuals with these characteristics might need tailored approaches to enhance their engagement.

**Level of evidence:**

Level V, retrospective descriptive study.

**Electronic supplementary material:**

The online version of this article (10.1007/s40519-020-00990-9) contains supplementary material, which is available to authorized users.

## Introduction

The weight management service attrition is a highly prevalent problem, affecting up to 80% of the patients with obesity [1–3]. The consequences are serious, not only in terms of the patient’s weight management but also because a failed weight loss attempt may be linked to feelings of frustration, discouragement, and learned helplessness [4]. The causes of drop-out have been extensively studied and the most frequently reported reasons have been: individual characteristics (younger age, higher BMI, health problems other than obesity, low socioeconomic status, job conditions or commitment, travel distance, lack of social support), weight loss-related aspects (unsuccessful weight-loss, previous attempts to lose weight, body shape concern, and high expectation of success), psychological factors (high levels of stress, depression, alexithymia, perceived sense of abandonment, higher binge eating score, and higher body dissatisfaction) [2, 3, 5–14]. However, other authors have failed to find relationships between personality traits and attrition [15].

Furthermore, although research in the field of engagement is growing, different terminology and criteria create ambiguity when comparing studies. Thus, dropping out or attrition, i.e. the decision to prematurely disengage from the service, can occur at various time points [4, 16]. Most studies on this topic make no distinction between early dropouts, late dropouts, and re-engage cases. Finally, many of these studies have been performed as part of randomized controlled trials or lifestyle intervention studies, where the conditions might be quite different from those in real-life [1, 7, 10–12, 17].

Owing to the importance of targeting patients with obesity to encourage their success in weight loss interventions, we aimed to study factors potentially implicated in early and late drop-out in a cohort of patients with obesity attending our obesity unit in a real-world setting.

## Methods

This was a retrospective cohort study. We analyzed all patients who attended their first visit at our obesity unit between 1 January 2017 and 31 December 2018. Eligible patients were those aged 18–75 years with a BMI ≥ 30 kg/m^2^ and < 45 kg/m^2^.

Our outpatient unit was part of the public health care system with minimal costs to the patients (Online Resource 1).

The exclusion criteria were: secondary causes of obesity (e.g. hypothalamic diseases, endocrine diseases, use of obesogenic drugs), previous bariatric surgery, known psychiatric disorders diagnosed by a MD (including known eating disorders), substance abuse within the last 12 months. We excluded the secondary causes of obesity since weight loss is strongly linked to the treatment of the primary cause; patients with a psychiatric illness were also not eligible because attrition and weight changes in these patients could be linked to the underlying disease and/or concomitant therapy, with the possible presence of many confounding factors.

Patients gave their informed consent for the collection and analysis of their data; the study was approved by the local Ethics Committee and was conducted in accordance with the Declaration of Helsinki principles.

### Definition of attrition

Patients who could not participate in a scheduled visit were provided with a new appointment. If the patient did not attend the visit, he/she was contacted by telephone and a new appointment was offered. Patient who did not attend a scheduled appointment and either did not answer the follow-up call or declared that he/she no longer wants to participate in the program was considered a dropout.

We defined patients who dropped out early as those who did not complete the initial 12 weeks of treatment (performed < 3 visits) and did not re-engage; we defined patients who dropped out late as those who disengaged from our weight loss program after the first 3 months and did not re-engage. Re-engaging patients were those who dropped out (at any time) and then requested to be included again in the program. Finally, we defined completers as those who missed no more than one meeting and attended the final meeting, while attenders were defined as those who missed > 1 meeting and attended the final meeting.

### Measurements

Weight (measured with the participants wearing light clothes and no shoes to the nearest 0.1 kg by a mechanical column scale, SECA model 711, Hamburg, Germany), height (measured to the nearest 0.1 cm with a Stadiometer, SECA 220 measuring rod, Hamburg, Germany), and waist and neck circumferences (measured by a plastic tape meter at the umbilicus level or under the cricoid cartilage, respectively) were assessed during each visit.

At baseline, all patients completed the following questionnaires:

- State-Trait Anxiety Inventory (STAI) for both trait (STAI-1) and state (STAI-2) anxiety [19];
- Binge Eating Scale (BES): a binge eating disorder was considered unlikely with scores 0–16, possible with scores 17–27 and likely with scores > 27 [20];
-Short-Form (SF)-36 health survey: used to measure general health-related quality of life; the SF-36 has different domains with scores ranging from 0 to 100 (higher values indicate a better quality of life, a value < 50 corresponds to low health status for each domain [21].
- The above psychometric questionnaires were checked and assessed by a trained psychologist.
- Arterial blood pressure values were measured on the left arm, in a sitting position, after at least 10 min of rest, with a mercury sphygmomanometer with appropriate cuff sizes (ERKA Perfect-Aneroid, Germany). Two measurements were taken with the arm supported at heart level and the values reported were the means of the two. Blood samples were collected after an overnight fast. Laboratory methods have been previously described [22].

### Statistical analyses

The *t*-test for paired data was applied to investigate within-group variations; the *t*-test for independent samples was performed to assess between-group differences. Because the distributions of the BDI score and anthropometric changes were skewed, the Mann–Whitney test was employed to evaluate between-group changes. A multiple logistic regression model, using attrition as the dependent variable, was used to evaluate the association between attrition and the variables of interest.

## Results

Out of the 381 patients who attended the first program visit at our obesity unit between 1/1/2017–31/12/2018, 250 met the inclusion criteria. Their characteristics are reported in Table 1. Out of all participants, 134 (53.6%) dropped out and did not complete the 12-month clinical course. Only 3 patients re-engaged after dropping our; they were considered together with the group of patients who dropped out. Among patients who completed the weight loss program, nobody missed more than 1 meeting; therefore, all of them were considered completers, and there were no attenders.

**Table 1:** Baseline characteristics of patients (left, all; central, by drop-out; right by early or late attrition)

Variables	All	Completers	Drop-outs	*P*	Early	Late	P*	P**	P***
Number	250	116	134		47	87			
Age (years)	50.6 ± 14.5	51.0 ± 14.2	50.3 ± 14.7	0.68	49.6 ± 15.8	50.6 ± 14.3	0.72	0.58	0.83
Males (%)	25.6	22.4	28.4	0.28	21.3	32.2	0.18	0.87	0.12
Residing in a rural area (%)	20.8	20.7	20.9	0.97	21.3	20.7	0.94	0.93	0.99
Active smoker (%)	13.6	13.8	13.4	0.93	21.3	9.2	0.05	0.23	0.32
Living alone (%)	42.4	37.9	46.3	0.18	59.6	39.1	0.023	0.011	0.87
Not working/retired (%)	44.4	41.4	47.0	0.37	51.1	44.8	0.49	0.26	0.62
Secondary schools/graduation (%)	62.0	63.8	60.5	0.59	55.3	63.2	0.37	0.31	0.93
Adverse life events (%)	41.6	41.4	41.8	0.95	48.9	37.9	0.22	0.38	0.62
Weight cycling (%)	58.4	57.8	59.0	0.85	66.0	55.2	0.23	0.33	0.71
Age of weight gain (years)	31.1 ± 14.9	31.3 ± 14.9	31.0 ± 15.0	0.86	30.6 ± 14.3	31.2 ± 15.4	0.83	0.78	0.95
Weight (kg)	98.6 ± 16.0	100.0 ± 15.9	97.4 ± 16.0	0.20	93.4 ± 13.6	99.6 ± 16.8	0.032	0.014	0.86
Height (cm)	163.2 ± 9.4	162.3 ± 9.0	163.9 ± 9.8	0.18	162.2 ± 9.4	164.9 ± 9.9	0.13	0.94	0.05
BMI (kg/m^2^)	36.9 ± 4.2	37.9 ± 4.2	36.1 ± 4.0	0.001	35.5 ± 3.8	36.5 ± 4.1	0.15	< 0.001	0.022
Waist circumference (cm)	116.9 ± 13.0	117.7 ± 12.0	116.2 ± 13.8	0.36	111.7 ± 12.8	118.6 ± 13.8	0.006	0.005	0.62
Neck circumference (cm)	38.9 ± 4.0	38.7 ± 3.8	39.1 ± 4.1	0.49	38.2 ± 3.9	39.6 ± 4.2	0.07	0.41	0.14
Systolic blood pressure (mmHg)	132.6 ± 12.6	132.1 ± 12.7	133.1 ± 12.5	0.52	131.8 ± 13.0	133.8 ± 12.3	0.37	0.90	0.33
Diastolic blood pressure (mmHg)	80.8 ± 6.9	80.5 ± 6.4	81.0 ± 7.4	0.61	80.6 ± 6.0	81.2 ± 8.0	0.65	0.96	0.52
Fasting glucose (mg/dL)	98.1 ± 26.2	97.1 ± 23.2	99.0 ± 28.5	0.55	97.9 ± 27.5	99.6 ± 29.2	0.75	0.83	0.49
Glycated hemoglobin (mmol/mol)	39.4 ± 10.2	39.9 ± 10.3	39.0 ± 10.1	0.48	38.4 ± 7.4	39.4 ± 11.4	0.58	0.34	0.71
Total cholesterol (mg/dL)	193.0 ± 40.3	194.3 ± 43.3	191.9 ± 37.7	0.64	195.3 ± 31.2	190.1 ± 40.9	0.45	0.89	0.48
HDL cholesterol (mg/dL)	52.0 ± 11.5	52.8 ± 12.0	51.3 ± 11.0	0.31	51.4 ± 10.0	51.3 ± 11.5	0.93	0.49	0.36
Triglycerides (mg/dL)	127.9 ± 74.0	132.0 ± 87.1	124.3 ± 60.4	0.41	123.6 ± 62.7	124.6 ± 59.4	0.92	0.55	0.50
Alanine aminotransferase (IU/L)	28.0 ± 18.1	28.0 ± 18.2	28.0 ± 18.1	0.98	29.7 ± 24.4	27.1 ± 13.7	0.42	0.61	0.71
Creatinine (mg/dL)	0.79 ± 0.22	0.79 ± 0.23	0.79 ± 0.20	0.99	0.78 ± 0.17	0.80 ± 0.22	0.71	0.82	0.88
*Psychological questionnaires*									
Beck Depression Inventory (BDI) score	7.0 (9.0)	6.0 (5.5)	8.0 (10.0)	0.010	11.0 (11.0)	8.0 (9.0)	0.07	0.004	0.10
Presence of moderate depression, BDI ≥ 20 (%)	7.2	6.9	7.5	0.86	12.8	4.6	0.09	0.23	0.49
STAI-1 (state anxiety) score	45.5 ± 11.4	45.3 ± 11.6	45.6 ± 11.2	0.81	46.8 ± 11.2	45.0 ± 11.2	0.38	0.45	0.86
Presence of state of anxiety (STAI-1 > 40) (%)	66.8	70.7	63.4	0.22	70.2	59.8	0.23	0.95	0.10
STAI-2 (anxious trait) score	47.1 ± 10.6	47.0 ± 10.1	47.2 ± 11.0	0.87	49.7 ± 11.6	45.9 ± 10.5	0.06	0.14	0.45
Presence of anxious trait (STAI-2 > 40) (%)	77.6	80.0	76.1	0.46	78.7	74.7	0.60	0.85	0.37
Binge eating scale (BES) score	31.6 ± 11.7	30.7 ± 10.9	32.4 ± 12.2	0.24	35.1 ± 13.1	31.0 ± 11.6	0.07	0.031	0.85
Probable binge eating (BES > 27) (%)	66.4	68.1	64.9	0.60	74.5	59.8	0.09	0.42	0.22
SF-36 (overall health state) score	51.6 ± 21.9	54.2 ± 22.0	48.5 ± 21.0	0.039	46.4 ± 23.2	49.7 ± 19.8	0.38	0.045	0.14
Low overall health status (SF-36-hs < 50)	45.2	39.7	50.0	0.10	59.6	44.8	0.10	0.021	0.46
SF-36 (health change) score	59.2 ± 26.9	59.8 ± 27.4	58.7 ± 26.6	0.76	61.0 ± 26.1	57.5 ± 26.9	0.48	0.80	0.56
Worse health change (SF-36-hc < 50)	15.6	13.8	17.2	0.46	17.0	17.2	0.97	0.60	0.50
SF-36 (mental health) score	56.1 ± 20.1	58.0 ± 21.7	54.4 ± 18.5	0.16	46.6 ± 16.9	58.6 ± 18.1	< 0.001	0.001	0.84
Low overall mental health (SF-36-mh < 50)	37.2	31.0	42.5	0.06	68.1	28.7	< 0.001	< 0.001	0.72

*Early dropouts *vs* late dropouts

**Early dropouts *vs* completers

***Late dropouts *vs* completers

There was a high prevalence of individuals who were living alone, who were not working and who reported adverse events during their life course (e.g., mourning, divorce, etc.). More than half of our patients reported weight cycling and weight gain at a young age (almost 20 years before their first visit to our unit). Even though patients with known psychiatric disorders were excluded from the analyses, a high prevalence of likely unknown anxiety and possible binge eating disorders were found among those included; these patients had declared the absence of any known disturbance (Table 1). Individuals who dropped out had lower BMIs, lower quality of overall health status and increased BDI (depression) scores. In a multiple regression model, BMI (inversely) and BDI score (directly) remained significantly associated with the risk of attrition (Table 2).

**Table 2:** Variables associated with the evaluated outcomes in a multiple logistic regression model

	OR	95% CI	*P*
*Dropout (yes/no)*			
Baseline BMI	0.90	0.84–0.96	< 0.001
BDI score	1.05	1.00–1.10	0.042
SF-36 overall health score	0.99	0.98–1.01	0.23
Early vs late dropout*			
Living alone	2.01	0.90–4.47	0.08
Baseline weight	0.97	0.95–1.00	0.05
Low SF-36 mental score	5.06	2.27–11.3	< 0.001
Early dropout vs completers**			
Living alone	2.25	1.02–4.97	0.042
Baseline BMI	0.83	0.75–0.92	< 0.001
BDI score	1.04	0.96–1.13	0.32
BES score	1.00	0.96–1.04	0.88
Low SF-36 overall health score	1.64	0.68–3.97	0.27
Low SF-36 mental score	3.17	1.17–8.59	0.022

*The same model was repeated by adjusting for baseline waist circumference instead of baseline weight and the results did not significantly change

**The same model was repeated by adjusting for baseline waist circumference instead of baseline BMI and the associations with low SF-36 mental score remained statistically significant (OR = 2.64; 95%CI 1.01–6.88; *p* = 0.045)

Changes in weight, BMI, waist, and neck circumference were statistically significant within both completers and dropouts (Online Resource 2). At the end of follow-up, in completers, weight loss was  – 6.75 ± 9.3 kg (median 6.33 kg) with a percent weight loss of 6.84 ± 8.9 (median  – 6.25%) and waist circumference reduction was  – 7.22 ± 9.0 (median  – 5.75 cm). Changes in anthropometric parameters at 3, 6, 9 months were greater in the completer group than in the dropout group, but between-group differences were not statistically significant. The percentages of patients treated with liraglutide were not different between these 2 groups (23.3% in the completer group and 16.4% in the dropout group, *p* = 0.17). Nobody was treated with orlistat. Very-low-calorie-diets were prescribed to 2.6% and 2.2% of completers and dropouts, respectively.

The characteristics of early and late dropouts are reported in Table 1. Patients who dropped out early had lower weights, waist circumferences and SF-36 scores for mental health and more frequently lived alone. Major differences were evident between the early dropout group and the completer group; the early dropout group had lower weights, lower BMIs, lower waist circumferences, lower SF-36 scores (both for overall health status and mental status), increased BDI scores and an increased proportion of individuals living alone. In a multiple logistic regression model, lower BMI, lower SF-36 mental score and living alone significantly increased the risk for early attrition (Table 2).

## Discussion

A high weight-loss program attrition rate was confirmed by the results from our study conducted in a community setting. Factors significantly associated with attrition were lower BMI and increased depression score. Approximately one-fifth of our patients dropped out early; those individuals had lower weights, reported decreased mental well-being, and more frequently lived alone.

### Attrition

Our finding of ~ 50% attrition is in line with the literature, since a high rate of attrition to weight-loss programs is common to most obesity units, with rates up to 80% after starting treatment [1–3, 13]. The dropout rates increased with the duration of follow-up, ranging from 20 to 30% in the first 4 months [5, 23] to 85% at 36 months [2, 24]. Accordingly, our attrition rates were 18.8%, 35.2%, 49.2% and 53.6% at 3, 6, 9, and 12 months, respectively.

Many different factors have been previously reported as potentially responsible for attrition with inconsistent results, including socioeconomic, demographic, weight-related and/or psychological conditions, such as depression, anxiety, alexithymia, presence of binge eating, body dissatisfaction, lack of motivation, unrealistic weight-loss expectations, low goal ownership, lower self-esteem, high levels of stress, and difficulty in coping with emotions [2, 3, 5–14, 25–30]. However, the data were highly contrasting [1, 14, 15], and the definitions of dropout greatly varied among the authors [4, 8, 16]. Furthermore, many of these studies were conducted within the context of randomized controlled trials or lifestyle intervention studies [1, 7, 10–12, 17, 30–32]; thus their results are not generalizable to a real-world setting.

We found a strong protective effect of BMI on attrition. Highly conflicting data are available on this topic [1]; no association [5, 7, 13, 15, 33, 34], a direct relationship [11, 25] and an inverse [23, 28, 35, 36] relationship have been reported. Individuals with higher BMI may be more motivated to lose weight; furthermore, healthcare professionals may pay more attention to the active involvement of patients with more severe obesity. Accordingly, the presence of comorbidity has been reported to cause greater adherence to weight-loss treatments, and completers were characterized by increased concern for one’s future or present health [13, 26, 35].

The weight loss achieved by our patients was in line with studies evaluating the effects of weight-loss programs in real clinical practice [6, 13, 15, 28, 37]. Weight loss was lower but not significantly different in late dropouts when compared to completers. Indeed, the differences between the two groups increased with time, and early weight loss has been suggested to be one of the factors motivating participants to continue the weight-loss program by many studies [6, 10, 13, 23, 28, 31, 38]. On the other hand, weight loss has been reported to be directly associated with the length of time that the individual remained in the weight-loss program [7]. We cannot exclude that the between-group difference could reach statistical significance in a larger sample; however, weight loss probably represents a less important factor in influencing attrition in our patients.

Our results suggest the importance of psychological distress (in particular, depression) in predicting attrition in weight-loss programs. Other authors have found that the presence of depression symptoms increased the risk of attrition [1, 9, 25, 31, 36]; an Italian study obtained the opposite result [35], and others reported no associations [14, 32].

Our participants had not been formally diagnosed with clinical depression; indeed, even mild scores of depressive symptoms could have an impact on attrition. Depression and attrition could be linked for several reasons, including patient lack of energy, impacting weight-loss compliance, the tendency to adopt maladaptive coping strategies (e.g., rumination, expressive suppression [39]), and the lower propensity to be engaged in the therapeutic alliance [40]. Further studies should investigate these clinically relevant topics.

### Early vs late attrition

Many studies had short follow-up (approximately 6 months) and/or did not differentiate between early and late attrition [7, 14, 15, 28, 33–35]. This makes their results difficult to compare. Older studies [16] suggested that individuals in the early attrition group had fewer prior attempts to lose weight, a higher external locus of control, and lower functional scores for depression, breadth of interest, organization, and responsibility. Accordingly, patients in our early dropout group showed specific characteristics that are suggestive of greater frailty, such as living alone and decreased mental well-being.

Living alone was a predictor of early dropout, in line with some authors [37], but differently from others [5, 13]. Living alone could be a proxy for multiple conditions including depression, the possible presence of other unreported psychiatric conditions, and being less motivated to cook healthy foods. However, more data would be necessary to draw more firm conclusions on this finding.

Decreased mental well-being was a significant risk factor for early attrition among our patients. Intriguingly, a questionnaire assessing many psychological aspects was more predictive of dropout risk than those assessing a single mental state, thus suggesting that the mental discomfort of those individuals was a complex mix of emotional, personality and social factors, rather than being characterized by specific symptomatology. This was in line with studies showing that scores combining depression, anxiety, social dysfunction, and somatic symptoms were better predictors of early attrition [8] and could justify the highly divergent results of previous studies that focused on only a few specific traits [1]. Greater mental stress can make it difficult to conduct many activities or handle different situations, such as traveling to the medical clinic, attending visits, interacting with specialists, comparisons with other participants, contact with new people, the change of one's lifestyle habits and tolerating the distress of potential failure. Our findings suggest the need for a broad assessment of mental health in these patients.

Early attrition leads to less weight loss [16]; psychological and psychiatric screening performed at baseline in all patients may be able to preemptively identify early dropouts. Patients experiencing greater mental stress might be better served by first addressing this burden before starting the weight loss program.

Even if late attrition is also regarded as a failure, its consequences seem to be less serious, since those individuals at least lost some weight, even though the weight loss was less than that achieved by the attender group. In a large sample of individuals mainly composed of participants who dropped out late, after 36 months of follow-up, the completer and attender groups showed a greater percentage of weight loss, but those in the dropout group who were satisfied or confident with their ability to reduce their weight reported further weight loss even without professional help, thus suggesting that not all attrition must be considered treatment failure [24]. It is of note that before starting treatment, patients tended to gain a considerable amount of weight [41], and even modest weight-loss could confer health benefits in the long term [42].

Strategies to enhance engagements may include increasing knowledge about the health problems linked to obesity, heightening awareness about the specific individual weight-loss barriers and obstacles and offering potential solutions, raising confidence about one’s control skills, acting on motivation in an individualized and realistic way, avoiding exclusively focusing on weight loss as an outcome but emphasizing the importance of healthy behaviors, better quality of life, and social functioning.

### Limitations

We did not assess other psychological factors, such as motivation to change, personality traits and coping abilities, which have been proven to be associated with attrition. Furthermore, we did not assess the reasons for attrition. Nevertheless, our evaluation was performed in a community setting, where the administration of many questionnaires to the patients is not feasible. The data were collected at baseline but may have changed over the course of treatment. Our sample size was small, and our study was limited to a single center with a specific weight-loss program, thus limiting the generalizability of our results. However, the post-hoc power analysis indicated that the study had a > 90% power to evaluate the between-group differences in BMI.

The strengths of our study were: the comprehensive assessment of multiple variables in all participants and the fact that all participants underwent an individual psychological assessment during which answers to the psychological questionnaires were verified and discussed instead of being simply self-reported, thus reducing the risk of either overestimating or underestimating psychopathology.

### Conclusions

Specific features seem to be related to the early weight-loss program attrition, suggesting the need for early evaluation and identification of those individuals at greatest risk to target tailored programs focused on improved long-term attendance.

## What is already known on this subject?

Attrition from weight management programs is frequent and contributes to obesity treatment failure.

## What your study adds?

Lower BMI and increased depression scores were associated with weight-loss program attrition in patients with obesity. Individuals in the early attrition group had lower weights, decreased mental well-being, and more frequently lived alone.

## Electronic supplementary material

Below is the link to the electronic supplementary material.

Supplementary file1 (DOCX 39 kb)

Supplementary file2 (DOCX 17 kb)

## Data Availability

The datasets generated and analyzed during the current study are not publicly available but will be available from the corresponding author upon request.

## References

[1] Moroshko I, Brennan L, O’Brien P (2011). Predictors of dropout in weight loss interventions: a systematic review of the literature. Obes Rev.

[2] Grossi E, Dalle Grave R, Mannucci E, Molinari E, Compare A, Cuzzolaro M, Marchesini G (2006). Complexity of attrition in the treatment of obesity: clues from a structured telephone interview. Int J Obes (Lond).

[3] Toth-Capelli KM, Brawer R, Plumb J, Daskalakis C (2013). Stage of change and other predictors of participant retention in a behavioral weight management program in primary care. Health Promot Pract.

[4] Nobles JD, Perez A, Skelton JA, Spence ND, Ball GD (2018). The engagement pathway: A conceptual framework of engagement-related terms in weight management. Obes Res Clin Pract.

[5] Honas JJ, Early JL, Frederickson DD, O’Brien MS (2003). Predictors of attrition in a large clinic-based weight-loss program. Obes Res.

[6] Elfhag K, Rössner S (2010). Initial weight loss is the best predictor for success in obesity treatment and sociodemographic liabilities increase risk for drop-out. Patient Educ Couns.

[7] Messier V, Hayek J, Karelis AD, Messier L, Doucet E, Prud’homme D, Rabasa-Lhoret R, Strychar I, (2010) Anthropometric, metabolic, psychosocial and dietary factors associated with dropout in overweight and obese postmenopausal women engaged in a 6-month weight loss programme: a MONET study. Br J Nutr 103:1230–1235. 10.1017/S0007114509993023 10.1017/S0007114509993023 19930768

[8] Michelini I, Falchi AG, Muggia C, Grecchi I, Montagna E, De Silvestri A, Tinelli C (2014). Early dropout predictive factors in obesity treatment. Nutr Res Pract.

[9] Benediktsdottir A, Halldorsson TI, Bragadottir GJ, Gudmundsson L, Ramel A (2016). Predictors of dropout and bariatric surgery in Icelandic morbidly obese female patients. Obes Res Clin Pract.

[10] Batterham M, Tapsell LC, Charlton KE (2016). Predicting dropout in dietary weight loss trials using demographic and early weight change characteristics: Implications for trial design. Obes Res Clin Pract.

[11] Goode RW, Ye L, Sereika SM (2016). Socio-demographic, anthropometric, and psychosocial predictors of attrition across behavioral weight-loss trials. Eat Behav.

[12] Sawamoto R, Nozaki T, Furukawa T, Tanahashi T, Morita C, Hata T, Komaki G, Sudo N (2016). Predictors of dropout by female obese patients treated with a group cognitive behavioral therapy to promote weight loss. Obes Facts.

[13] Perna S, Spadaccini D, Riva A (2018). A path model analysis on predictors of dropout (at 6 and 12 months) during the weight loss interventions in endocrinology outpatient division. Endocrine.

[14] Altamura M, Porcelli P, Fairfield B, Malerba S, Carnevale R, Balzotti A, Rossi G, Vendemiale G, Bellomo A (2018). Alexithymia predicts attrition and outcome in weight-loss obesity treatment. Front Psychol.

[15] Dalle Grave R, Calugi S, Compare A, El Ghoch M, Petroni ML, Colombari S, Minniti A, Marchesini G (2015). Personality, attrition and weight loss in treatment seeking women with obesity. Clin Obes.

[16] Miller BML, Brennan L (2015). Measuring and reporting attrition from obesity treatment programs: A call to action!. Obes Res Clin Pract.

[17] Mutsaerts MQ, Kuchenbecker WKH, Mol BW, Land JA, Hoek A (2013). Dropout is a problem in lifestyle intervention programs for overweight and obese infertile women: a systematic review. Hum Reprod.

[18] Beck AT (1967). Depression.

[19] Spielberger CD (1983). State-trait anxiety inventory for adults: sampler set: Manual, Test.

[20] Gormally J, Black S, Daston S, Rardin D (1982). The assessment of binge eating severity among obese persons. Addict Behav.

[21] McHorney CA, Ware JE, Raczek AE (1993). The MOS 36-Item Short-Form Health Survey (SF-36): II. Psychometric and clinical tests of validity in measuring physical and mental health constructs. Med Care.

[22] Bo S, Ponzo V, Ciccone G, Evangelista A, Saba F, Goitre I, Procopio M, Pagano GF, Cassader M, Gambino R (2016). Six months of resveratrol supplementation has no measurable effect in type 2 diabetic patients. A randomized, double blind, placebo-controlled trial. Pharmacol Res.

[23] Packianathan I, Sheikh M, Boniface D, Finer N (2005). Predictors of programme adherence and weight loss in women in an obesity programme using meal replacements. Diabetes Obes Metab.

[24] Dalle Grave R, Melchionda N, Calugi S, Centis E, Tufano A, Fatati G, Fusco MA, Marchesini G (2005). Continuous care in the treatment of obesity: an observational multicentre study. J Intern Med.

[25] Teixeira PJ, Going SB, Sardinha LB, Lohman TG (2005). A review of psychosocial pre-treatment predictors of weight control. Obes Rev.

[26] Dalle Grave R, Calugi S, Molinari E, Petroni ML, Bondi M, Compare A, Marchesini G, QUOVADIS Study Group (2005). Weight loss expectations in obese patients and treatment attrition: an observational multicenter study. Obes Res.

[27] Dalle Grave R (2006). Factors associated with attrition in weight loss programs. Int J Behav Consult Ther.

[28] Colombo O, Ferretti VV, Ferraris C, Trentani C, Vinai P, Villani S, Tagliabue A (2014). Is drop-out from obesity treatment a predictable and preventable event?. Nutr J.

[29] Dalle Grave R, Calugi S, El Ghoch M (2018). Are personality characteristics as measured by the temperament and character inventory (TCI) associated with obesity treatment outcomes? A systematic review. Curr Obes Rep.

[30] Huisman S, Maes S, De Gucht VJ, Chatrou M, Haak HR (2010). Low goal ownership predicts drop-out from a weight intervention study in overweight patients with type 2 diabetes. Int J Behav Med.

[31] Fabricatore AN, Wadden TA, Moore RH, Butryn ML, Heymsfield SB, Nguyen AM (2009). Predictors of attrition and weight loss success: results from a randomized controlled trial. Behav Res Ther.

[32] Bradshaw AJ, Horwath CC, Katzer L, Gray A (2010). Non-dieting group interventions for overweight and obese women: what predicts non-completion and does completion improve outcomes?. Public Health Nutr.

[33] De Panfilis C, Torre M, Cero S, Salvatore P, Dall’Aglio E, Marchesi C, Cabrino C, Aprile S, Maggini C, (2008) Personality and attrition from behavioral weight-loss treatment for obesity. Gen Hosp Psychiatry 30:515–520. 10.1016/j.genhosppsych.2008.06.003 10.1016/j.genhosppsych.2008.06.003 19061677

[34] Minniti A, Bissoli L, Di Francesco V, Fantin F, Mandragona R, Olivieri M, Fontana G, Rinaldi C, Bosello O, Zamboni M (2007). Individual versus group therapy for obesity: comparison of dropout rate and treatment outcome. Eat Weight Disord.

[35] Inelmen EM, Toffanello ED, Enzi G, Gasparini G, Miotto F, Sergi G, Busetto L (2005). Predictors of drop-out in overweight and obese outpatients. Int J Obes (Lond).

[36] Clark MM, Niaura R, King TK, Pera V (1996). Depression, smoking, activity level, and health status: pretreatment predictors of attrition in obesity treatment. Addict Behav.

[37] Busetto L, Mazza M, Salvalaio S, De Stefano F, Marangon M, Calò E, Sampietro S, Enzi G (2009). Obesity treatment in elderly outpatients: predictors of efficacy and drop-out. Eat Weight Disord.

[38] Wadden T, Letizia K, Wadden TA, VanItallie TB (1992). Predictors of attrition and weight loss in patients treated by moderate to severe caloric restriction. Treatment of the Seriously Obese Patient.

[39] Joormann J, Stanton CH (2016). Examining emotion regulation in depression: a review and future directions. Behav Res Ther.

[40] Pompili M, Venturini P, Palermo M, Stefani H, Seretti ME, Lamis DA, Serafini G, Amore M, Girardi P (2013). Mood disorders medications: predictors of nonadherence - review of the current literature. Expert Rev Neurother.

[41] Blomquist KK, Barnes RD, White MA, Masheb RM, Morgan PT, Grilo CM (2011). Exploring weight gain in year before treatment for binge eating disorder: a different context for interpreting limited weight losses in treatment studies. Int J Eat Disord.

[42] Jensen MD, Ryan DH, Apovian CM (2014). 2013 AHA/ACC/TOS Guideline for the Management of Overweight and Obesity in Adults. Circulation.

