# Supplementary material for: Predictors of attrition from a weight loss program. A study of adult patients with obesity in a community setting
Source: Eat Weight Disord. 2020 Aug 20;26(6):1729–36. doi: 10.1007/s40519-020-00990-9 (PMC8292291; doi:10.1007/s40519-020-00990-9)
Supplement: Supplementary file 1 — Supplementary file1 (DOCX 39 kb) [file 40519_2020_990_MOESM1_ESM.docx]

**Eating and Weight Disorders**

**Predictors of attrition from a weight loss program. A study of adult patients with obesity in a community setting**

Valentina Ponzo^1^, Elena Scumaci^1^, Ilaria Goitre^1^, Guglielmo Beccuti^1^, Andrea Benso^1,2^, Sara Belcastro^1^, Chiara Crespi^1^, Franco De Michieli^1,2^, Marianna Pellegrini^1^, Paola Scuntero^2^, Enrica Marzola^3^, Giovanni Abbate-Daga^3^, Ezio Ghigo^1,2^, Fabio Broglio^1,2^, Simona Bo^1,2^

^1^ Department of Medical Sciences, University of Torino, Italy

^2^ Center for the Treatment of Diabetes and Metabolic Diseases, “Città della Salute e della Scienza” Hospital of Torino, Italy

^3^ Department of Neuroscience, University of Torino, Italy

**Corresponding author:** Simona Bo, [simona.bo@unito.it](mailto:simona.bo@unito.it)

**Online Resource 1. Description of our weight loss program**

Our weight loss program included at least 6 meetings over 12 months (**Figure 1**). In specific cases, supplementary visits could be scheduled (e.g. in the case of prescription of a very-low-calorie diet or in the case of drug prescription). During the first visit, after being welcomed by the nurses, the patients completed sociodemographic, psychological and food questionnaires, then they received a group session of nutritional education; subsequently they were visited by the endocrinologist, the psychologist and the dietician. During the following scheduled visits, participants received assessments by the dietician, the endocrinologist or both, according to the program; supplementary visits were provided for patients with specific needs.

We provided verbal and written dietary, exercise and behavior recommendations to all participants during individual and group visits. All patients received three group-sessions (two conducted by a dietician plus a psychologist, one conducted by a psychologist alone) and three individual visits with trained dieticians. An individually prescribed diet that followed the Mediterranean diet composition was provided (45-55% carbohydrates, <10% sugars, 30% fats, <10% saturated fats, 15-25% proteins, 20-30 g fiber) with an energy restriction ranging from 500 to 1000 kcal, based on the individual caloric requirements and usual assumption. During group- and one-to-one meetings, simple recommendations were given, by focusing on practical lifestyle tips (about food composition, portion control, identification of common dietary mistakes, how to identify high-sugar, high-fat and high-calorie foods, how to choose high-fiber foods, how to reduce the intakes of salt, food/beverages with added sugar, options for dining-out and healthy shopping). Patients were invited to register their weekly food intake in a food diary to increase their awareness.

Similarly, verbal, and written advice on exercise was given, mainly by suggesting moderate activity, such as brisk walks for at least 150 minutes/week plus 30 minutes/week of exercise against resistance. Practical recommendations on how to increase daily exercise and include physical movement into habitual activities were provided both in the individual and group visits. All patients received a brief written guide containing all the lifestyle recommendations and behavioral changes suggested.

Patients received at least four individual visits with trained endocrinologists, who evaluated their health status, comorbidity, and risk factors, performed a detailed anamnestic data collection and physical examination, evaluated their blood parameters, and, if needed, suggested further specific exams and prescribed drugs. In Italy, in 2017-2018, only orlistat and liraglutide were available for prescription. Furthermore, the MDs provided clear explanations about the medical risks of obesity and the benefits of a healthy lifestyle in the prevention/control of those risks, established reasonable weight objectives to minimize excessive expectations by the patients and explained the mechanisms implicated in weight regain and hunger/satiety control and the rationale of maintaining adequate muscle mass.

Two psychologists, specialized in obesity treatment, individually visited all patients at baseline and discussed with them the completed psychological questionnaires; their weight-loss motivation, expectations, perceptions, and self-confidence; and their satisfaction level with their current weight. Furthermore, they participated to the group sessions with the dieticians and held an additional group session about the importance of self-monitoring, goal setting, stimulus control, problem-solving, relapse prevention, and self-manage during hunger moments or social gatherings (e.g. during parties, at the restaurant, etc). If psychological reinforcement or support was needed, individual meetings (up to eight) were proposed during the weight-loss program.

Trained nurses welcomed all participants, explained the 12-months program and followed-up the patients by 3-4 individual meetings in which they reinforced the patients’ motivation, by analyzing and sharing their strengths, weaknesses, and opportunities.

The weight-loss program usually lasts 12 months; however, it can be extended with further visits according to the needs of the individual. The weight loss target was individualized and discussed with the patient. Generally, a 10% weight loss at 6 months was proposed. However, more or less stringent goals were suggested based on individual needs. All patients were followed for at least 1 year, even if they had achieved their weight loss goal before the end of the program.

**Figure 1. Weight-loss program of our Obesity Unit**
