# Supplementary material for: Predictors of attrition from a weight loss program. A study of adult patients with obesity in a community setting
Source: Eat Weight Disord. 2020 Aug 20;26(6):1729–36. doi: 10.1007/s40519-020-00990-9 (PMC8292291; doi:10.1007/s40519-020-00990-9)
Supplement: Supplementary file 2 — Supplementary file2 (DOCX 17 kb) [file 40519_2020_990_MOESM2_ESM.docx]

**Eating and Weight Disorders**

**Predictors of attrition from a weight loss program. A study of adult patients with obesity in a community setting**

Valentina Ponzo^1^, Elena Scumaci^1^, Ilaria Goitre^1^, Guglielmo Beccuti^1^, Andrea Benso^1,2^, Sara Belcastro^1^, Chiara Crespi^1^, Franco De Michieli^1,2^, Marianna Pellegrini^1^, Paola Scuntero^2^, Enrica Marzola^3^, Giovanni Abbate-Daga^3^, Ezio Ghigo^1,2^, Fabio Broglio^1,2^, Simona Bo^1,2^

^1^ Department of Medical Sciences, University of Torino, Italy

^2^ Center for the Treatment of Diabetes and Metabolic Diseases, “Città della Salute e della Scienza” Hospital of Torino, Italy

^3^ Department of Neuroscience, University of Torino, Italy **Corresponding author:** Simona Bo, [simona.bo@unito.it](mailto:simona.bo@unito.it)

**Online Resource 2. Anthropometric changes during follow-up**

|  | **Completers** | **Late dropouts** | **P** |
| --- | --- | --- | --- |
| **3 months** |  |  |  |
| Number | 116 | 87 |  |
| Weight | 97.7±16.1** | 97.7±17.0** | 0.99 |
| Median weight change | -1.85 | -1.50 | 0.29 |
| Median percent weight loss | 1.82 | 1.31 | 0.28 |
| BMI | 37.0±4.4** | 35.8±4.2** | 0.06 |
| Median BMI change | -0.68 | -0.52 | 0.23 |
| Waist circumference | 114.8±14.1** | 116.3±12.4** | 0.42 |
| Median waist circumference change | -2.00 | -1.50 | 0.86 |
| Neck circumference | 39.1±8.5 | 38.7±4.4** | 0.69 |
| Median neck circumference change | -0.50 | -0.50 | 0.55 |
| **6 months** |  |  |  |
| Number | 116 | 46 |  |
| Weight | 94.9±16.8** | 97.2±16.8** | 0.45 |
| Median weight change | -4.69 | -3.06 | 0.14 |
| Median percent weight loss | 5.00 | 3.25 | 0.19 |
| BMI | 35.9±4.6** | 35.3±4.8** | 0.45 |
| Median BMI change | -1.81 | -1.17 | 0.11 |
| Waist circumference | 112.8±11.9** | 114.3±17.7** | 0.54 |
| Median waist circumference change | -6.00 | -3.75 | 0.60 |
| Neck circumference | 37.7±3.8** | 38.7±3.7** | 0.12 |
| Median neck circumference change | -1.00 | -0.85 | 0.91 |
| **9 months** |  |  |  |
| Number | 116 | 11 |  |
| Weight | 94.0±17.2** | 92.8±15.6** | 0.82 |
| Median weight change | -5.75 | -3.60 | 0.58 |
| Median percent weight loss | 5.73 | 3.64 | 0.59 |
| BMI | 35.6±5.0** | 34.1±4.9** | 0.33 |
| Median BMI change | -2.02 | -1.47 | 0.50 |
| Waist circumference | 111.6±11.9** | 114.1±10.8* | 0.49 |
| Median waist circumference change | -6.00 | -2.50 | 0.32 |
| Neck circumference | 37.3±3.6** | 38.7±3.3** | 0.55 |
| Median neck circumference change | -1.00 | -1.00 | 0.29 |
| **12 months** |  |  |  |
| Number | 116 | - |  |
| Weight | 93.3±18.0** | - |  |
| Median weight change | 6.33 | - |  |
| Median percent weight loss | -6.25 | - |  |
| BMI | 35.3±5.3** | - |  |
| Median BMI change | -2.32 | - |  |
| Waist circumference | 110.5±12.2** | - |  |
| Median waist circumference change | -5.75 | - |  |
| Neck circumference | 37.0±3.8** | - |  |
| Median neck circumference change | -1.00 | - |  |

*p<0.05 and **P<0.01 by t-test for paired data (comparison with baseline values)

Median values were compared by Mann-Whitney U-test
